# Analysis of the effectiveness of the application of pelvic floor rehabilitation exercise and the factors influencing its self-efficacy in postoperative patients with cervical cancer

**DOI:** 10.3389/fonc.2023.1118794

**Published:** 2023-05-09

**Authors:** Xichun Li, Ling Liu, Jinhui He, Jue Yan, Ying Wang

**Affiliations:** ^1^ Department of Rehabilitation, Chengfei Hospital, Chengdu, China; ^2^ Department of Rehabilitation, Bayi Orthopaedic Hospital, Chengdu, China; ^3^ Department of Obstetrics and Gynecology, Chengdu Seventh People’s Hospital, Chengdu, China; ^4^ Department of Rehabilitation, Southwest Medical University Affiliated Hospital of Traditional Chinese Medicine, Luzhou, China; ^5^ Department of Oncology, Sichuan Provincial People’s Hospital, Chengdu, China

**Keywords:** cervical cancer, radical surgery, pelvic floor rehabilitation exercise, self-efficacy, influencing factors

## Abstract

**Objective:**

To analyze the application effect of pelvic floor rehabilitation exercise in postoperative patients with cervical cancer and the factors influencing their self-efficacy.

**Methods:**

120 postoperative patients with cervical cancer from January 2019 to January 2022 from the Department of Rehabilitation, Aeronautical Industry Flying Hospital, Bayi Orthopaedic Hospital and Southwest Medical University Affiliated Hospital of Traditional Chinese Medicine, and the Department of Obstetrics and Gynecology, Chengdu Seventh People’s Hospital, and the Department of Oncology, Sichuan Provincial People’s Hospital were selected for the study. They were divided into routine group (n=44, applied routine care) and exercise group (n=76, applied routine care + pelvic floor rehabilitation exercise) according to the different perioperative care programs. The perioperative indicators, bladder function recovery rate and urinary retention incidence, urodynamic indicators, and pelvic floor distress inventory-short form 20 (PFDI-20) scores were compared between the 2 groups. The general data, PFDI-20 scores and broome pelvic muscle self-efficacy scale (BPMSES) scores of patients in the exercise group were investigated and analyzed individually to investigate the factors influencing the self-efficacy of patients with pelvic floor rehabilitation exercise after cervical cancer surgery.

**Results:**

The time of first anal exhaust, urine tube retention and hospitalization after surgery were shorter in the exercise group than in the routine group (P<0.05). The bladder function grade I rate after surgery was more in the exercise group than in the routine group, and the urinary retention incidence was lower than that in the routine group (P<0.05). At 2 weeks after exercise, bladder compliance and bladder detrusor systolic pressure were higher in both groups than before exercise, and they were higher in the exercise group than in the routine group (P<0.05). There was no significant difference in urethral closure pressure within and between the two groups (P>0.05). At 3 months after surgery, the PFDI-20 scores were higher in both groups than before surgery, and the exercise group was lower than the routine group (P<0.05).The BPMSES score for the exercise group was (103.33 ± 9.16). Marital status, residence and PFDI-20 scores were influential factors in the self-efficacy level of patients undergoing pelvic floor rehabilitation exercise after cervical cancer surgery (P<0.05).

**Conclusion:**

Implementing pelvic floor rehabilitation exercise for postoperative patients with cervical cancer can speed up the recovery of pelvic organ function and reduce the occurrence of postoperative urinary retention. Marital status, residence and PFDI-20 scores were influential factors in the self-efficacy level of patients undergoing pelvic floor rehabilitation exercise after cervical cancer surger, medical staff need to incorporate these clinical features to provide targeted nursing interventions to enhance patient compliance with training and improve postoperative survival quality.

## 1 Introduction

Cervical cancer is the fourth common cancer in women worldwide, and is also the only cancer with clear cause and preventable in the world at present (1). However, due to the influence of work pressure, living habits and other factors, the age of diagnosis of cervical cancer tends to be younger, which seriously affects women’s physical and mental health (2, 3). According to the data of International Agency for Research on Cancer (IARC) of WHO in 2020, cervical cancer is the tenth largest cancer in China (4), and 57% of patients with the onset age of less than 45 years old (5). Radical resection of cervical cancer combined with pelvic lymph node dissection is the standard scheme for clinical treatment of early stage cervical cancer, but this method has a wide range of resection, which can cause varying degrees of damage to bladder muscles, nerves innervating the bladder, pelvic floor ligaments, etc. (6, 7). It has been reported that among the pelvic organ dysfunctions triggered by postoperative cervical cancer, the prevalence of lower urinary tract dysfunction can reach 70%-85% (8) and sexual dysfunction can reach 66.67% (9), in addition to triggering various negative emotions (10). Therefore, taking effective measures to prevent pelvic organ dysfunction is important to improve the rehabilitation outcome and the quality of survival of patients. Biofeedback electrical stimulation is a kind of pelvic floor rehabilitation therapy. It applies electric current of different frequencies and pulse widths to patients through vaginal electrodes to stimulate passive contraction of muscles, exercise pelvic floor muscles, improve the resting tension of pelvic floor muscles, restore the coordinated relaxation and contraction function of pelvic floor muscles, and strengthen the contraction of urethral sphincter and urinary control ability (11). Kegel exercise is a training method invented by Kegel, an American obstetrician and gynecologist, to strengthen pelvic floor muscles. By consciously contracting and relaxing vaginal, urethral and perianal muscles, it can promote the recovery of bladder function (12). In recent years, some domestic scholars (13) advocated that the pelvic floor rehabilitation exercise of biofeedback electrical stimulation combined with Kegel exercise should be used as a first-line treatment option for pelvic floor dysfunction diseases after cervical cancer surgery, but the safety of its clinical application and patient compliance remain controversial. Self-efficacy, as a positive psychological belief, highlights an individual’s efficient and positive response to the emergence of health problems, and patients with higher self-efficacy have higher adherence to treatment (14). Based on the above, this study investigated the application effect of pelvic floor rehabilitation exercise in postoperative patients with cervical cancer and analyzed the factors influencing the self-efficacy of patients who implemented pelvic floor rehabilitation exercise in order to develop a targeted and practical pelvic floor functional rehabilitation intervention program to enhance the compliance and survival quality of pelvic floor muscle training in postoperative survivors of cervical cancer.

## 2 Materials and methods

### 2.1 Research subjects

120 postoperative patients with cervical cancer from January 2019 to January 2022 from the Department of Rehabilitation, Aeronautical Industry Flying Hospital, Bayi Orthopaedic Hospital and Southwest Medical University Affiliated Hospital of Traditional Chinese Medicine, and the Department of Obstetrics and Gynecology, Chengdu Seventh People’s Hospital, and the Department of Oncology, Sichuan Provincial People’s Hospital were selected for the study. Inclusion criteria: age 15-45 years; patients with histologically confirmed IAI (+) LVSI, IA2, IB1, IB2 and IIA1 stage disease (FIGO staging of cervical cancer (15)); patients who underwent type B/C radical hysterectomy (Querleu-Morrow classification (16)); no tumor metastasis; postoperative survival >12 months; complete postoperative follow-up data; normal cognitive and motor functions; all surgical and care protocols of the study were discussed and determined by the principal investigator and sponsor of each center, and a standardized surgical and care process was established, and the procedures were performed after approval by the ethics committee. Exclusion criteria: preoperative history of pelvic or urological surgery; preoperative radiotherapy or chemotherapy; preoperative combined urinary retention or moderate or higher urinary incontinence; combined neuromuscular diseases; combined psychiatric or psychological disorders; combined other malignant tumors, severe somatic diseases or systemic diseases. They were divided into routine group (n=44) and exercise group (n=76) according to the different perioperative care programs. There was no significant difference (P > 0.05) between the two groups in terms of general information such as age, body mass index, FIGO staging, and Querleu-Morrow classification, which were comparable (**Table 1**).

**Table 1:** General information for both groups.

Itmes	Routine group (n=44)	Exercise group (n=76)	t/χ^2^	P
Age (years old)	31.77 ± 5.94	33.70 ± 6.27	1.656	0.100
Body mass index (kg/m^2^)	23.24 ± 1.92	22.87 ± 2.04	0.978	0.330
educational level			2.053	0.562
primary school and below	9 (20.45)	12 (15.79)		
junior high school	10 (22.73)	20 (26.32)		
vocational high school/technical secondary school	18 (40.91)	25 (32.89)		
college and above	7 (15.91)	19 (25.00)		
FIGO staging			0.950	0.917
IAI (+) LVSI	3 (6.82)	6 (7.89)		
IA2	5 (11.36)	12 (15.79)		
IB1	12 (27.27)	18 (23.68)		
IB2	7 (15.91)	9 (11.85)		
IIA1	17 (38.64)	31 (40.79)		
Querleu-Morrow classification			0.151	0.697
Type B	10 (22.73)	15 (19.74)		
Type C	34 (77.27)	61 (80.26)		

### 2.2 Care programs

(1) Routine group was applied with routine care. The content included: Giving patients perioperative psychological counseling to relieve their psychological pressure. Postoperatively, strengthen urinary tract care, ensure smooth catheterization, prevent urinary tract infection, and timely observation and recording of urine volume, urine color and properties. Patients were instructed to perform preoperative rehabilitation exercise training and postoperative bladder function rehabilitation training, etc., for 8 weeks of continuous training after surgery.
(2) Exercise group was applied routine care (as above) + pelvic floor rehabilitation exercise. The latter consisted of 2 parts: ① Pelvic floor biofeedback electrical stimulation exercise: On the 3rd postoperative day, the patient was treated with Urostym biofeedback electrostimulator (Though, Canada, model: MyoTrace Pro180) in a lateral position after emptying the bowels, disinfecting the perianal area with saline, thoroughly relaxing, lubricating with paraffin oil, gently inserting the electrodes into the anus, giving bioelectrical stimulation at a frequency of 20 Hz and a current of 40~75 mA, adjusted according to the patient’s feeling of muscle throbbing without pain, for 30 min, once a day, 7 days as a course of treatment. ② Kegel exercise training: The patient lied flat, adjusted breathing to calm, contracted the gluteal muscles to lift the anus upward during inhalation, tightened the urethra, vagina and anus, held for 5~10s, relaxed during exhalation, repeated at 5~10s intervals. The patient stood with hands crossed on both shoulders, toes at 90°, heels medially as wide as the armpits, clenched hard for 5-10s, relaxed on exhalation, and repeated at 5-10s intervals. The patient stood with hands crossed on both shoulders, toes at 90°, heels medially as wide as the armpits, clamp firmly for 5-10s, relaxed on exhalation, and repeated at 5-10s intervals. The patient squatted down, pulled the bed rail with both hands, opened the feet shoulder-width apart, and slowly did the “squat-stand-squat” movement. Exercise instruction: Produce a video on Kegel exercise training methods, introduce the purpose and significance of Kegel exercise training to patients and their families, and demonstrate standardized movements on site before surgery. Establish a patient wechat group and send the video to the wechat group to guide and supervise the patients to perform Kegel exercise training. Exercise frequency: lying down, standing, squatting 3 movements repeated 20 ~ 30 times for a group. 3 to 5 sets of 15 to 30 min per day before surgery. After the patient’s vital signs were stabilized after surgery, appropriate exercise methods and exercise frequency were selected according to the patient’s physical condition and recovery, to the extent that the patient was slightly sweating and did not feel pain and fatigue. 1~2 sets for the first time, 2~3 sets for the 2nd and 3rd times, 4~5 sets per day from the 4th time onwards. 7 days for 1 session, 8 weeks of continuous training after surgery.

### 2.3 General information collection

The content covers the patient’s age, marital status, education level, occupation, residence, number of children, pathological stage, average monthly family income, and medical insurance type, etc.

### 2.4 Analysis of indicators

(1) Perioperative indicators: Time of first anal exhaust, urine tube retention and hospitalization after surgery were compared between 2 groups.
(2) Bladder function recovery rate and urinary retention incidence: Residual urine volume <50 ml with good recovery of bladder function was defined as grade I; residual urine volume 50-100 ml with slightly poor recovery of bladder function was defined as grade II; residual urine volume >100 ml with poor recovery of bladder function was defined as grade III; and extremely difficult urination with no recovery of bladder function was defined as grade IV. The rate of each grade of bladder function and the incidence of urinary retention after surgery were compared between 2 groups of patients.
(3) Urodynamic indicators: Bladder compliance, urethral closure pressure and bladder detrusor systolic pressure were compared between 2 groups of patients before and 2 weeks after exercise to assess their urination dynamics and quality.
(4) Pelvic floor distress inventory-short form 20 (PFDI-20) scores: PFDI-20 scores were compared between 2 groups of patients before and 3 months after surgery. The PFDI-20 consists of 3 subscales of urination (POPDI-6), defecation (CRADI-8) and pelvic organ prolapse (UDI-6) with a total of 20 questions, with scores ranging from 0 to 100 for each subscale, and the scores were proportional to the severity of symptoms. The Cronbach’s α coefficients of each scale ranged from 0.790 to 0.879, and the retest reliability ranged from 0.776 to 0.818, indicating strong correlation between the questions of the scale and good stability of the scale.
(5) Broome pelvic muscle self-efficacy scale (BPMSES) scores: BPMSES scores were calculated for patients in the exercise group before exercise. BPMSES consists of 2 dimensions of expected self-efficacy (14 entries) and expected outcome (9 entries) with a total of 23 entries, each with a score of 0 to 10, and the scores were proportional to the level of self-efficacy, specifically, a total score of <50 is poor self-efficacy, 50-70 is general self-efficacy, and >70 is good self-efficacy. The Cronbach’s α coefficients for the total scale and each dimension were 0.845 to 0.941, and the retest reliability was 0.841 to 0.935.

### 2.5 Survey method

The investigators were trained and assessed by a unified survey, and a unified instruction language was used to explain to the patients about the cautions of filling out the questionnaires, and if there were people with low literacy level or language communication barriers, the questionnaires could be read and then explained by the person who issued them or with the help of other ethnic minorities to translate on their behalf.

### 26 Statistical analysis

Data analysis was performed with SPSS 22.0 software. Count data were shown as % and χ^2^ test was taken, measurement data were expressed as ( ± s), one-way ANOVA was taken for comparison between multiple groups, and t-test was taken for comparison between 2 groups. The factors influencing the self-efficacy of pelvic floor rehabilitation exercise were analyzed by multiple stepwise linear regression. Differences were considered statistically significant at P<0.05.

## 3 Results

### 3.1 Perioperative indicators

**Figure 1:**
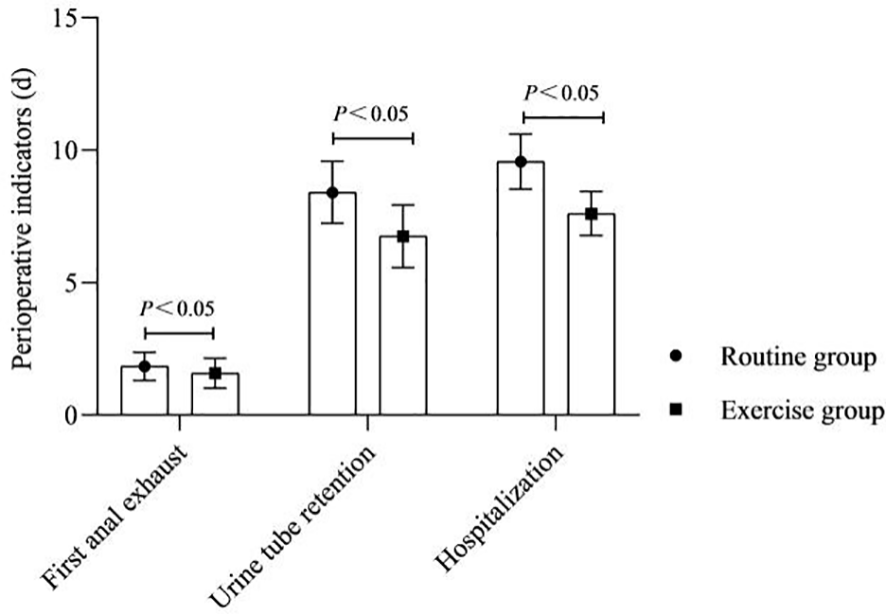
Perioperative indicators.

As shown in **Figure 1**. The time of first anal exhaust, urine tube retention and hospitalization after surgery were shorter in the exercise group than in the routine group (P<0.05).

### 3.2 Bladder function recovery rate and urinary retention incidence

**Figure 2:**
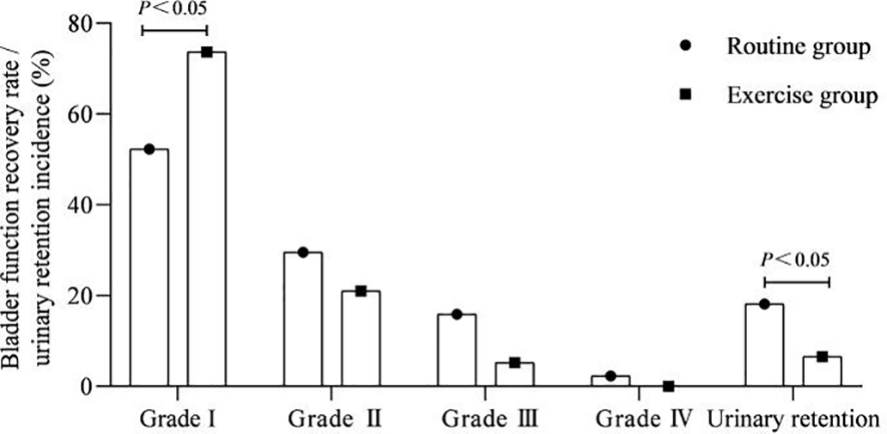
Bladder function recovery rate and urinary retention incidence.

As shown in **Figure 2**. The bladder function grade I rate after surgery was more in the exercise group than in the routine group, and the urinary retention incidence was lower than that in the routine group (P<0.05).

### 3.3 Urodynamic indicators

**Figure 3:**
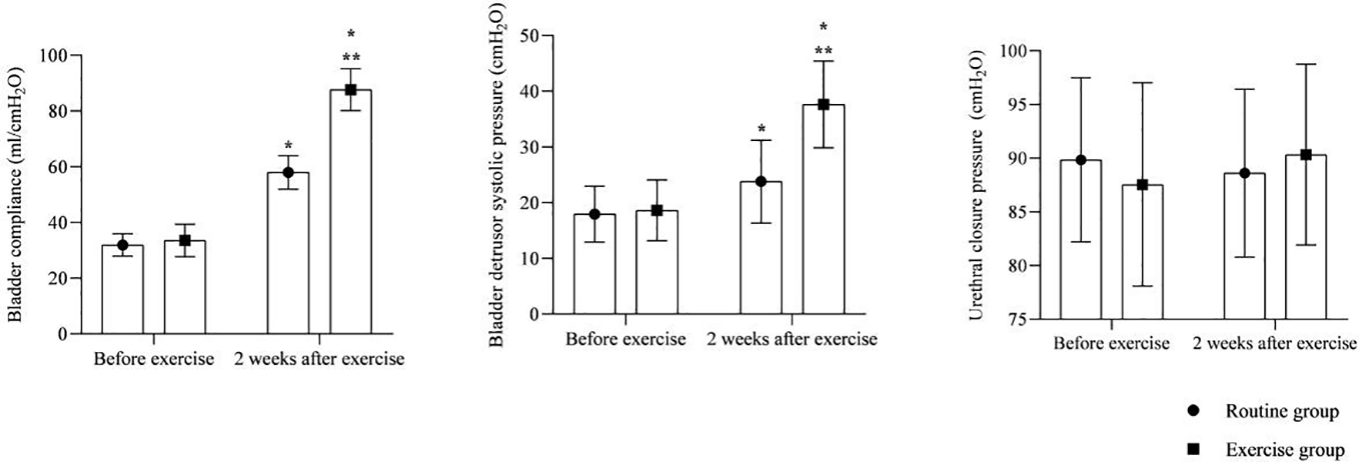
Urodynamic indicators. * is compared with the same group before exercise, ** is compared with the routine group 2 weeks after exercise, P<0.05.

As shown in **Figure 3**. At 2 weeks after exercise, bladder compliance and bladder detrusor systolic pressure were higher in both groups than before exercise, and they were higher in the exercise group than in the routine group (P<0.05). There was no significant difference in urethral closure pressure within and between the two groups (P>0.05).

### 3.4 PFDI-20 scores

**Figure 4:**
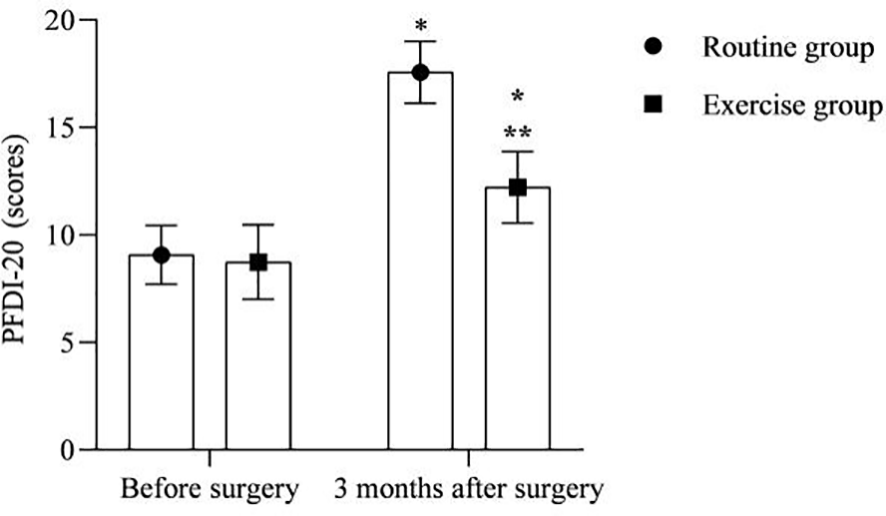
PFDI-20 scores. * is compared with the same group before surgery, ** is compared with the routine group 3 months after surgery, P<0.05.

As shown in **Figure 4**. At 3 months after surgery, the PFDI-20 scores were higher in both groups than before surgery, and the exercise group was lower than the routine group (P<0.05).

### 3.5 BPMSES scores

**Figure 5:**
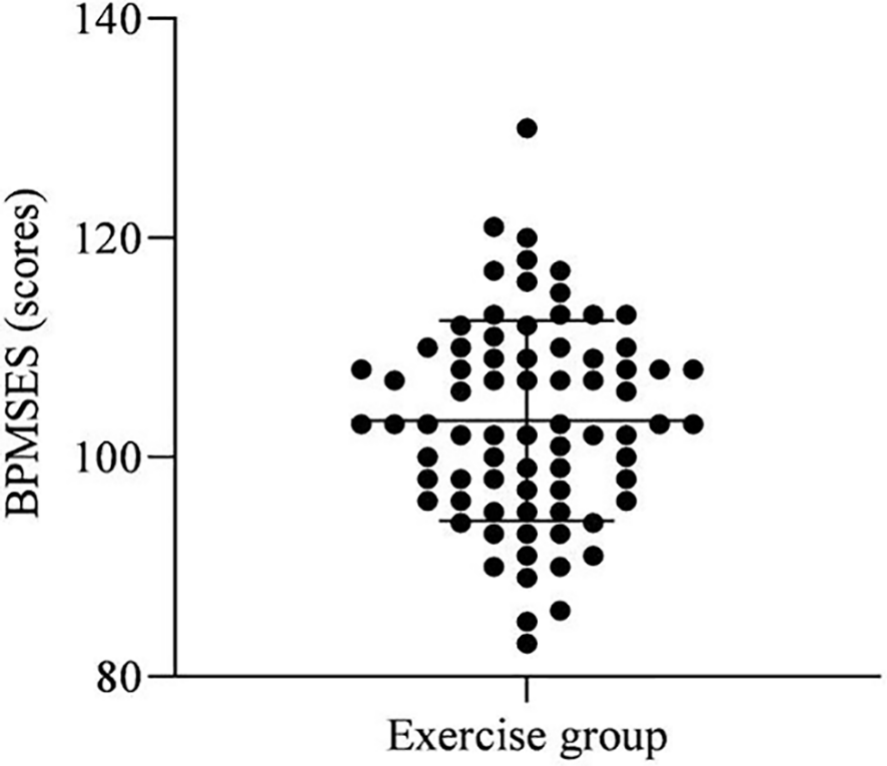
BPMSES scores.

As shown in **Figure 5**. The BPMSES score for the exercise group was (103.33 ± 9.16).

### 3.6 One-way ANOVA on self-efficacy of 76 patients with postoperative pelvic floor rehabilitation exercise for cervical cancer

As shown in **Table 2**. Self-efficacy level of patients with postoperative pelvic floor rehabilitation exercise for cervical cancer was related to patients’ age, marital status, occupation, and residence (P<0.05).

**Table 2:** One-way ANOVA on self-efficacy of 76 patients with postoperative pelvic floor rehabilitation exercise for cervical cancer (scores).

Factors	n	Self-efficacy score	F/t	P
Age			36.210	<0.001
15~<25 years old	11	122.42 ± 8.45		
25~<35 years old	30	94.94 ± 10.47		
35~45 years old	35	89.63 ± 12.48		
Marital status			16.550	><0.001
unmarried	17	118.85 ± 11.05		
married	52	108.28 ± 7.36		
divorced	4	99.42 ± 8.24		
widowed	3	86.77 ± 9.52		
Education level			0.373	0.772
primary school and below	12	101.42 ± 12.40		
junior high school	20	102.80 ± 8.64		
vocational high school/technical secondary school	25	104.50 ± 8.63		
college and above	19	104.58 ± 10.71		
Occupation			9.492	<0.001
farmers	11	95.41 ± 10.45		
workers	8	100.84 ± 9.24		
cadres	12	114.87 ± 6.45		
housewives	9	104.08 ± 6.33		
professional and technical personnel	15	116.50 ± 9.47		
retirees	2	84.83 ± 12.47		
service workers	10	101.14 ± 8.41		
others	9	108.97 ± 8.69		
Residence			6.806	<0.001
rural	34	95.87 ± 11.58		
urban	42	110.79 ± 7.42		
Number of children			2.282	0.086
0 pcs	15	108.55 ± 7.23		
1 pcs	30	103.78 ± 9.54		
2 pcs	24	103.43 ± 10.44		
≥3 pcs	7	97.56 ± 9.47		
Pathological stage			0.322	0.726
phase IA	18	102.47 ± 6.28		
phase IB	27	102.97 ± 12.03		
phase IIA	31	104.55 ± 9.08		
Average monthly family income			0.848	0.500
0~<1000 yuan	7	99.14 ± 8.33		
1000~<2000 yuan	14	101.26 ± 7.59		
2000~<3000 yuan	29	102.08 ± 11.37		
3000~<4000 yuan	21	103.76 ± 11.45		
≥5000 yuan	5	109.41 ± 9.70		
Medical insurance type			0.709	0.496
medical insurance	54	106.15 ± 12.47		
rural cooperative medical care	18	103.60 ± 9.02		
self-funded	4	100.24 ± 10.28		

### 3.7 Multiple stepwise linear regression analysis of self-efficacy in 76 patients with postoperative pelvic floor rehabilitation exercise for cervical cancer

As shown in **Tables 3**, **4**. Marital status, residence and PFDI-20 scores were influential factors in the self-efficacy level of patients undergoing pelvic floor rehabilitation exercise after cervical cancer surgery (P<0.05).

**Table 3:** Assignment Table.

Factors	Assignment
Age	15~<25 years old=1, 25~<35 years old=2, 35~45 years old=3
Marital status	unmarried=1, married, divorced=3, widowed=4
Occupation	farmers=1, workers=2, cadres=3, housewives=4, professional and technical personnel=5, retirees=6, service workers=7, others=8
Residence	rural=1, urban=2
PFDI-20 scores	continuous variable

**Table 4:** Multiple stepwise linear regression analysis of self-efficacy in 76 patients with postoperative pelvic floor rehabilitation exercise for cervical cancer.

Factors	β	t	P	Adjustment R^2^	F
Marital status	-0.167	-3.120	0.004	13.674	23.860
Residence	0.121	2.128	0.035	17.201	16.458
PFDI-20 scores	0.112	2.050	0.038	17.953	14.344

## 4 Discussion

Patients with cervical cancer undergoing radical cervical cancer surgery are more effective enough to remove the lesion and reduce the residual rate of the lesion, but because the surgery itself requires the dissection of the uterine ligament and its surrounding supporting tissues, while the relative position and function of the rectum, bladder, and neurovascularity are abnormally altered, causing a double destruction of the structural integrity and function of the pelvic floor, which can lead to the occurrence of postoperative pelvic organ dysfunction (17).

In this study, we found that pelvic floor rehabilitation exercise with biofeedback electrical stimulation combined with Kegel exercise promoted the recovery of pelvic organ function in postoperative patients with cervical cancer. After exercise, the patients’ anal venting and urinary catheter retention time were shortened, and the recovery of pelvic organ dysfunction was accelerated, which shortened the patients’ rehabilitation process and hospitalization time. Analysis of the reasons may be that biofeedback electrical stimulation enhances the contractility of pelvic floor muscle and the motor function of levator ani muscle and striated muscle around the urethra through the electrical stimulation conduction of neuromuscle, promotes the recovery of pelvic floor nerve conduction and neural network reconstruction, creates conditions for the recovery of bladder muscle neural function, and is conducive to the development of Kegel exercise for patients with cervical cancer after surgery (18). Simultaneously, it inhibits to some extent the bladder nerve excitation conduction, improves the sensitivity of bladder filling, and increases the action of the urethral sphincter, therefore reducing the occurrence of postoperative urinary retention (19). Kegel exercise training, on the other hand, targeted contraction-diastolic training of the pelvic floor muscle groups by the patient autonomously with the levator ani muscle mainly, can effectively improve bladder muscle relaxation and promote the recovery of the patient’s bladder neck support, which reduces postoperative urinary retention (20).

Kegel exercise requires the patient to accurately identify and appreciate the relevant pelvic floor muscles, and without proper instruction, more than 50% of Kegel exercises are irregular or even completely wrong (21). Meanwhile, patients with cervical cancer are accompanied by severe negative emotions, and the trauma of radical surgery can add to their painful experience, and the effectiveness of self-exercise of the pelvic floor muscles is lower in patients. In this study, the purpose and meaning, exercise methods and precautions of Kegel exercise after cervical cancer surgery were repeatedly introduced through video education, live demonstration and Wechat support, and different frequencies of Kegel exercise were implemented according to the patients’ physical condition and recovery, with the aim of standardizing Kegel exercise for postoperative patients with cervical cancer and promoting the development of their compliance behavior. From the results, the BPMSES score of patients in the exercise group of this study was (103.33 ± 9.16), indicating that the implementation of pelvic floor rehabilitation exercise with biofeedback electrical stimulation combined with Kegel exercise can promote the development of self-efficacy of pelvic floor muscle training in patients after cervical cancer surgery. Analysis of the influential factors affecting patients’ self-efficacy for pelvic floor rehabilitation exercise revealed that marital status, residence, and PFDI-20 scores were all influential factors in the level of self-efficacy of patients for pelvic floor rehabilitation exercise after cervical cancer surgery. Analyzing the reasons for the above situation, marital status: The self-efficacy of pelvic floor rehabilitation training was higher in unmarried patients than in married, divorced and widowed patients, probably related to the fact that such patients are not married and have children, and the need for future marriage is more prominent, so they have stronger confidence to improve their postoperative quality of life and do their best to return to normal family life, with a view to minimizing the postoperative effects. In contrast, most of the married, divorced and widowed patients are married and have given birth to children, with the declining need for marriage and childbirth, the level of inner pain is relatively low, and therefore the motivation for rehabilitation exercise is lacking. In the prediction model of Jiang et al. (22), the marital status of patients with cervical cancer is also related to their prognosis, suggesting that targeted interventions by medical personnel based on this clinical feature may also be crucial to improving the survival chances of patients. Residence: The self-efficacy of pelvic floor rehabilitation exercise was higher in urban patients than in rural patients, probably because most rural patients came from remote areas with poor economic and medical conditions and were usually busy with their lives lacking attention to themselves. Some surveys (23–25) showed a lack of knowledge related to cervical cancer prevention and treatment among rural women, and various effective means should be taken to provide more extensive health education to rural women and the public. Consequently, different forms of health education should be provided to enhance the patients’ knowledge of the disease and improve their confidence and compliance with rehabilitation training. PFDI-20 score: Postoperative patients with cervical cancer will have different degrees of pelvic floor dysfunction, with an incidence of about 63.93%, among which the incidence of urinary dysfunction and pelvic organ prolapse can be as high as 70-85%, and the incidence of defecation dysfunction is about 20% (26). The comfort of patients with these symptoms is greatly affected. In particular, patients with bowel and gas incontinence, because of the special smell will inevitably cause embarrassment, worry about the surrounding partners, friends and relatives cast strange eyes, so easy to produce negative pessimism, the more negative emotions, the worse self-confidence, easy to worry about not being respected and treated differently, so most patients in this category will appear out of social conditions, further reducing their pelvic floor functional status.

## 5 Conclusion

Implementing pelvic floor rehabilitation exercise for postoperative patients with cervical cancer can speed up the recovery of pelvic organ function and reduce the occurrence of postoperative urinary retention. Marital status, residence and PFDI-20 scores were influential factors in the self-efficacy level of patients undergoing pelvic floor rehabilitation exercise after cervical cancer surger, medical staff need to incorporate these clinical features to provide targeted nursing interventions to enhance patient compliance with training and improve postoperative survival quality.

## Data availability statement

The datasets presented in this study can be found in online repositories. The names of the repository/repositories and accession number(s) can be found in the article/supplementary material.

## Ethics statement

This study was approved by the ethics committee of our hospital. The patients/participants provided their written informed consent to participate in this study.

## Author contributions

The first author is XL, he is mainly responsible for collecting experimental data. LL, JH and JY mainly carried out data analysis and participated in writing papers, Enhance the readability of articles. YW is the corresponding authors, and she is responsible for ensuring that the descriptions are accurate. All authors contributed to the article and approved the submitted version.
